# Composition and Function of Decellularized Human Lung Extracellular Matrix from Congenital Pulmonary Airway Malformation

**DOI:** 10.3390/jcm15176742

**Published:** 2026-08-30

**Authors:** Yanan Li, Ping Yang, Miao Yuan, Xinglong Zhu, Shengqiang Mao, Ying Yang, Menglin Yao, Fei Chen, Yanyan Zhou, Ji Bao, Chang Xu, Yi Li

**Affiliations:** 1 Department of Respiratory and Critical Care Medicine, State Key Laboratory of Respiratory Health and Multimorbidity, Institute of Respiratory Health, Frontiers Science Center for Disease-Related Molecular Network, West China Hospital, Sichuan University, Chengdu 610041, China; noodleli@outlook.com (Y.L.); msq941028@163.com (S.M.); yangying2380@163.com (Y.Y.); yaomenglin8388@wchscu.cn (M.Y.); 2 Department of Pediatric Surgery, West China Hospital, Sichuan University, Chengdu 610041, China; yuanmiao@wchscu.cn; 3 Department of Pathology, Institute of Clinical Pathology, Key Laboratory of Transplant Engineering and Immunology, West China Hospital, Sichuan University, Chengdu 610041, China; yangping1109@163.com (P.Y.); zhuxinglong@stu.scu.edu.cn (X.Z.); chenfei82207747@163.com (F.C.); zhouyanyan@wchscu.cn (Y.Z.); baoji@scu.edu.cn (J.B.)

**Keywords:** congenital pulmonary airway malformation, extracellular matrix, decellularization, proteomics, lung development

## Abstract

**Background**: Congenital pulmonary airway malformation (CPAM) is a rare developmental disorder characterized by cystic lung lesions, yet its extracellular matrix (ECM) composition remains poorly understood. This study employed decellularization and data-independent acquisition (DIA) proteomics to compare ECM profiles between cystic (CPAM) and histologically normal non-diseased (ND) regions from the lungs of four patients. **Results**: The decellularized scaffolds retained their native architecture with minimal residual DNA (<50 ng/mg). Proteomic analysis revealed 431 differentially expressed proteins (DEPs), with 171 upregulated and 260 downregulated in CPAM. Key findings revealed CPAM-specific enrichment of collagens (COL4A6, COL4A2, COL10A1, COL21A1 and PIIINP), glycoproteins (SPP1, FRAS1, FREM1, FREM2, LTBP1 and FBLN7), ECM regulators (TENM2, ROR2 and OMD), and ECM-affiliated proteins (ANXA7), alongside downregulation of glycoproteins (VASN and ABI3BP), proteoglycans (PODN and MXRA7), ECM regulators (SCARA5, PAPPA, SAA4, CPXM1, CTSC, THY1, SERPINA6/A1/D1, BSG, LYVE1, ITIH4 and CD44), and ECM-affiliated proteins (LGALSL). Pathway analysis highlighted the dysregulation of TGF-β, PI3K–AKT, and mTOR signaling in CPAM and aberrant ECM–cell interactions in pathogenesis. We also evaluated their functional properties and investigated the impact of ECM-based hydrogels on recellularization. **Conclusions**: These findings provide a comprehensive proteomic atlas of CPAM ECM alterations, offering insights into disease mechanisms and potential therapeutic targets.

## 1. Introduction

Congenital pulmonary airway malformation (CPAM), previously termed congenital cystic adenomatoid malformation (CCAM), is a rare but complex congenital pulmonary disorder [1]. It is pathologically characterized by the presence of multiple cystic lesions comprising disorganized airspaces that maintain continuity with the tracheobronchial tree and are vascularized by bronchial artery-derived vessels [2]. With an incidence of 0.094–0.4% of births in recent years [1,3], the pathogenesis of CPAM, which manifests during fetal development (typically prior to the 20th gestational week), remains incompletely understood. Current evidence suggests that disrupted epithelial–mesenchymal interactions during pulmonary morphogenesis correlate with mesenchymal cell hyperplasia and impaired apoptosis, ultimately resulting in cystic lung lesion formation [2]. The spatiotemporal distribution of key transcription factors such as ACTA2, SOX2, and SOX9 orchestrates normal human lung branching morphogenesis [4]. Disruption of these developmental programs has been implicated in CPAM pathogenesis. In fact, the first comprehensive transcriptomic analysis of congenital lung malformations revealed dysregulated expression of genes related to the Ras and PI3K-AKT-mTOR signaling pathways, with evidence of a cell-autonomous defect in the lesion epithelium [5]. Diagnosis relies on prenatal ultrasonography and postnatal imaging techniques, including chest radiography and computed tomography (CT), particularly in patients who present with pediatric respiratory distress or acute infectious complications. In addition, CPAM predisposes patients to multiple clinical risks, including recurrent pulmonary infections, respiratory distress, failure to thrive, and potential malignant transformation [3,6]. While surgical resection in infancy or childhood remains the standard treatment for symptomatic patients and is often curative, it does not completely eliminate the risk of long-term pulmonary complications, such as recurrent infections, air trapping, and potential exercise intolerance. Therefore, these patients may benefit from long-term follow-up to monitor for such sequelae.

The lung is a complex organ that has remarkable physiological sophistication and cellular heterogeneity and plays a pivotal role in respiratory function. Current research has elucidated the multifaceted role of the extracellular matrix (ECM), which extends beyond its traditional structural support function to include critical regulatory roles in organ homeostasis, regeneration, and health [7]. The protein composition of the ECM is highly diverse and includes proteoglycans, matrix-associated growth factors, and various secreted factors that orchestrate tissue architecture and cellular processes such as proliferation, differentiation, and maturation. Dysregulation of the intricate interplay between the ECM and resident cells often results in detrimental health effects [8,9]. In the developing lung, ECM dynamics are essential for proper branching morphogenesis and alveolarization. During embryonic development, temporal and spatial changes in ECM composition, particularly in collagens, elastin, and proteoglycans, provide the physical cues and biochemical signals that guide epithelial progenitor cell proliferation, migration, and differentiation [10,11]. A landmark conceptual advance identifying ECM dynamics as an emerging hallmark of aging was recently reported [12,13]. The biomechanical properties of ECM and their consequences for cellular homeostasis are recognized as key drivers of organismal aging, alongside established hallmarks such as genomic instability, epigenetic alterations, and cellular senescence. Disruption of these finely tuned ECM remodeling processes has been implicated in a spectrum of congenital and acquired lung disorders. Among several chronic lung diseases, chronic obstructive pulmonary disease (COPD) and idiopathic pulmonary fibrosis (IPF) have been the most characterized, demonstrating that alterations in the ECM structure and composition significantly contribute to aberrant tissue regeneration and disease pathogenesis [9,14,15,16]. In COPD, ECM degradation predominates, driven by an imbalance between matrix metalloproteinases (MMPs) and their tissue inhibitors (TIMPs). This results in fragmentation of elastin and collagens, leading to alveolar wall destruction and permanent airspace enlargement. The resulting ECM fragments themselves act as damage-associated molecular patterns (DAMPs), perpetuating chronic inflammation through toll-like receptor signaling. Meanwhile, in IPF, excessive ECM accumulation, particularly of fibrillar collagens and fibronectin, is a hallmark of disease. This pathological fibrosis creates a stiffened, mechanically abnormal microenvironment that promotes fibroblast-to-myofibroblast differentiation and sustains a vicious cycle of matrix deposition via mechanotransduction pathways such as YAP/TAZ and TGF-β. Consequently, elucidating the structural and bioactive ECM components within the lung under both normal and disease conditions is critical.

Recent advancements in proteomic technologies have enabled comprehensive characterization of tissue-specific ECM composition. In pulmonary research, mass spectrometry-based analysis of decellularized lungs from animals or cadaveric humans has facilitated semiquantitative profiling of lung ECM components [17]. Current studies have demonstrated that decellularized lungs preserve a substantial proportion of the native ECM matrix composition from the lungs of COPD and IPF patients, including structural ECM proteins such as type I and type III collagens (COL1 and COL3) and bioactive ECM proteins such as laminins, proteoglycans and glycoproteins [18,19]. Furthermore, data-independent acquisition (DIA) proteomics can detect both low- and high-abundance proteins, providing more comprehensive proteome coverage. However, comprehensive proteomic characterization of the lesional CPAM ECM remains unexplored. In this study, decellularized lungs from patients with CPAM were assessed via DIA proteomics, and the alterations were compared, providing valuable insight into the global ECM composition in normal and diseased lungs in CPAM.

## 2. Materials and Methods

### 2.1. Human Lung Tissue Collection

This study was approved by the Ethics Committee of West China Hospital, Sichuan University (project identification code: 2019-1082). All the subjects provided informed consent. Lung resection specimens were obtained from CPAM patients who were diagnosed via CT scan and underwent surgery at West China Hospital. All enrolled patients met the predefined inclusion criteria and had no evidence of active infection at the time of surgery. Inclusion criteria: (1) diagnosis of CPAM confirmed by postnatal chest CT imaging prior to surgical resection; (2) availability of paired tissue samples from both the cystic lesion and adjacent histologically normal-appearing lung parenchyma; (3) sufficient tissue quantity for decellularization and downstream proteomic analysis. Exclusion criteria: (1) evidence of active pulmonary infection at the time of surgery; (2) history of preoperative antibiotic therapy within 4 weeks prior to surgery; (3) prior surgical intervention or biopsy of the CPAM lesion; (4) diagnosis of other congenital lung malformations, e.g., bronchogenic cyst, congenital lobar emphysema; (5) presence of major associated congenital anomalies or syndromic diagnoses; (6) insufficient tissue quality or quantity for decellularization and proteomic analysis. The diagnosis and classification of CPAM were confirmed via histological analysis after surgical resection according to the Stocker classification. For each CPAM patient, we collected tissues from the cystic area (CPAM, *n* = 4) and histologically normal-appearing adjacent tissue surrounding the lesion as a within-patient comparator (ND, *n* = 4). Clinical information on the patient demographics is presented in Table 1.

### 2.2. Human Lung Decellularization

The lung tissues were immersed in optimal cutting temperature (OCT) and rapidly frozen. Twenty-micrometer-thick sections were cut and gently collected. The sections were then decellularized as previously described [20]. In brief, tissue sections were rinsed with phosphate-buffered saline (PBS) and double-distilled water (ddH_2_O). Decellularization was performed with 0.1% Triton-X 100, 0.1% sodium lauryl ether sulfate (SLES), 0.1% Triton-X 100 and ddH_2_O every 6 h. Decellularization efficiency was assessed via hematoxylin and eosin (H&E) staining, and residual DNA was measured via a DNeasy Blood & Tissue Kit (Qiagen, Germantown, MD, USA) according to the manufacturer’s protocols. The morphology of the ECM was also determined by scanning electron microscopy (SEM). Electron micrographs of lung cross-sections were obtained at 5.0 kV and ×600 magnification via a Hitachi S-4800 SEM (Hitachi, Chiyoda City, Japan). The samples were stored at −80 °C until future use.

### 2.3. Astral DIA Proteomic Analysis of Decellularized ECM

Protein from decellularized ECM was extracted from tissue samples using SDT lysis buffer (4% sodium dodecyl sulfate (SDS), 100 mM dithiothreitol (DTT), and 100 mM Tris-HCl, pH 8.0), heated for 3 min and further ultrasonicated. The samples were subsequently centrifuged at 16,000× *g* for 15 min. The supernatants were collected and quantified with a BCA protein assay kit (BeyoTime, Shanghai, China) according to the manufacturer’s protocols.

Protein digestion was performed via the FASP method with detergent, DTT and iodoacetamide (IAA) in UA buffer. The protein suspension was digested with trypsin (Promega, Chuo City, Japan) at a ratio of 50:1 overnight at 37 °C. The peptide mixtures were collected by centrifugation at 16,000× *g* for 15 min and desalted with C18 StageTip for further LC–MS analysis. The concentrations of the redissolved peptides were determined using the OD280 via a Nanodrop One device (Thermo, Waltham, MA, USA).

LC–MS/MS was performed on an Orbitrap Astral mass spectrometer coupled with a Vanquish Neo UHPLC system (Thermo Fisher Scientific, Waltham, MA, USA). Peptides from each sample were loaded onto a column (50 cm Low-Load µPAC™ Neo HPLC Column, Thermo Scientific, Waltham, MA, USA) at a flow rate of 2.2 μL/min. The RP-HPLC mobile phase A was 0.1% formic acid in water, and B was 0.1% formic acid in 80% acetonitrile. The peptides were eluted over 8 min with a linear gradient of buffer B at 1.25 μL/min. The linear gradient was set as follows: 0–0.1 min, linear gradient from 4% to 6% buffer B; 0.1–1.1 min, linear gradient from 6% to 12% buffer B; 1.1–4.3 min, linear gradient from 12% to 25% buffer B; 4.3–6.1 min, linear gradient from 25% to 45% buffer B; 6.1–6.5 min, linear gradient from 45% to 99% buffer B; and 6.5–8 min, buffer B maintained at 99%. The eluted peptides were analyzed on an Orbitrap Astral mass spectrometer. The DIA method consists of a survey scan from 380 to 980 *m*/*z* at a resolution of 240,000 with an AGC target of 500% and a 5 ms injection time. The DIA MS/MS scans were acquired by Astral from 150–2000 *m*/*z* with a 2 *m*/*z* isolation window and with AGC targets of 500% and 3 ms injection times. The normalized collision energy was set to 25, and the cycle time was 0.6 s. The spectra of the full MS scan and DIA scan were recorded in profile and centroid types, respectively. The DIA-MS data were analyzed via DIA-NN 1.8.1, and the bioinformatics results were analyzed.

### 2.4. Bioinformatics Analysis

To annotate the sequences, information was extracted from UniProtKB/Swiss-Prot, Kyoto Encyclopedia of Genes and Genomes (KEGG), and Gene Ontology (GO). GO and KEGG enrichment analyses were carried out with Fisher’s exact test, and FDR correction for multiple testing was also performed. The GO terms were grouped into three categories: biological process (BP), molecular function (MF), and cellular component (CC). Enriched GO and KEGG pathways were nominally statistically significant at a Fisher’s exact test level of q < 0.05. The construction of protein–protein interaction (PPI) networks was also conducted via the STRING database with the Cytoscape v3.8.1 software.

For the validation data, public genomics data from the Gene Expression Omnibus (GEO) database were downloaded. The dataset GSE190620 was used, and differential expression was assessed.

### 2.5. Lung Decellularized ECM Hydrogel Evaluation

Lung decellularized ECM hydrogel was prepared as previously described on ice. One gram of lung decellularized ECM was enzymatically digested in 50 mL of 2 g l−1 porcine pepsin (Sigma-Aldrich, Saint Louis, MO, USA) in 0.01 M HCl at a stirring rate of 60 rpm for 48 h at room temperature. The digestion was terminated by titration to pH 7.2–7.4 with 0.1 M NaOH. Next, the neutralized lung decellularized ECM pregel solution was diluted with 10× PBS at a ratio of 9:1 (*v*/*v*) for later use.

The lung decellularized ECM hydrogel was diluted and added into 24-well cell culture plates, and the plates were incubated at 37 °C for 1 h for hydrogel gelation. The plates were washed with PBS. Human umbilical vein endothelial cells (HUVECs) were seeded onto plates and cultivated in an incubator with saturated humidity and 5% CO_2_ at 37 °C. The morphology, viability and proliferation of the cells after 3 d were evaluated using rhodamine phalloidin solution (100 nM, Cytoskeleton, Denver, CO, USA) and α-tubulin (Proteintech, Rosemont, IL, USA, 11224-1-AP, 1:200), a cellular viability calcein AM/propidium iodide fluorescent stain (Solarbio, Beijing, China), and the cells were imaged using an Axio Observer D1/AX10 cam HRC fluorescence microscope (Carl Zeiss, Göttingen, Germany).

### 2.6. Statistics

ECM proteins can be further classified into subgroups as collagens, ECM glycoproteins, ECM regulators, ECM-affiliated proteins, proteoglycans, or secreted factors. Individual ECM proteins were analyzed and normalized as the relative abundance of spectral hits for each respective ECM protein type relative to the total spectral hits of ECM proteins within each respective sample. For proteomics and pathway enrichment analyses (GO and KEGG), Fisher’s exact test was used with FDR correction, and a level of q < 0.05 was considered significant. In individual protein-level analyses, paired t-tests were used for comparisons. A level of *p* < 0.05 was considered significant. All the data were analyzed using SPSS 25.0 and organized via GraphPad Prism 9. The data are presented as the means ± standard errors of the mean (SEMs).

## 3. Results

### 3.1. Assessment of Decellularized Lungs

**Figure 1:**
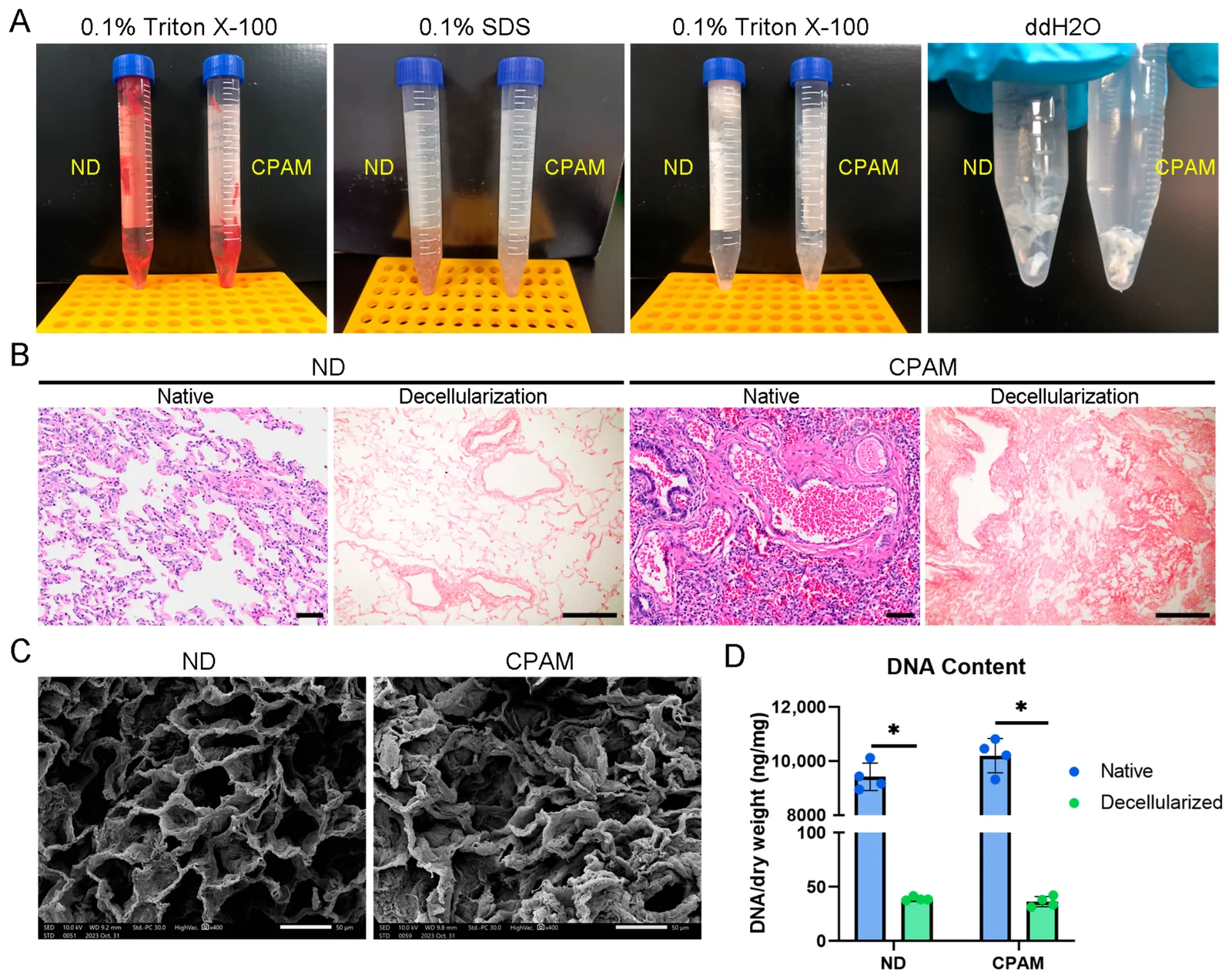
Morphology and characteristics of the decellularized lung scaffolds. (**A**) Schematic of decellularized lung processing. (**B**) H&E staining of native (non-decellularized) human patient lungs showing the lung morphology of CPAM patients and ND lungs. (**C**) Representative image of decellularized CPAM and ND lungs. (**D**) Relative DNA content in native lung and decellularized lung scaffolds from CPAM and normal ND lungs. The bars indicate the means ± SEMs. * *p* < 0.05 versus the native group. Scale bar = 50 μm.

Visual inspection revealed that the detergents produced white and translucent lung ECM after decellularization (Figure 1A). The decellularized ND and CPAM lungs retained much of the histological appearance of the native lungs (Figure 1B). The SEM results revealed that the ultrastructure of the ECM from decellularized CPAM lungs presented with cystic lesions similar to those observed in native CPAM lungs (Figure 1C). Minimal residual DNA (<50 ng/mg) was observed both quantitatively and qualitatively in the lungs after decellularization (Figure 1D).

### 3.2. A Comprehensive Map of Decellularized Lungs from the CPAM

**Figure 2:**
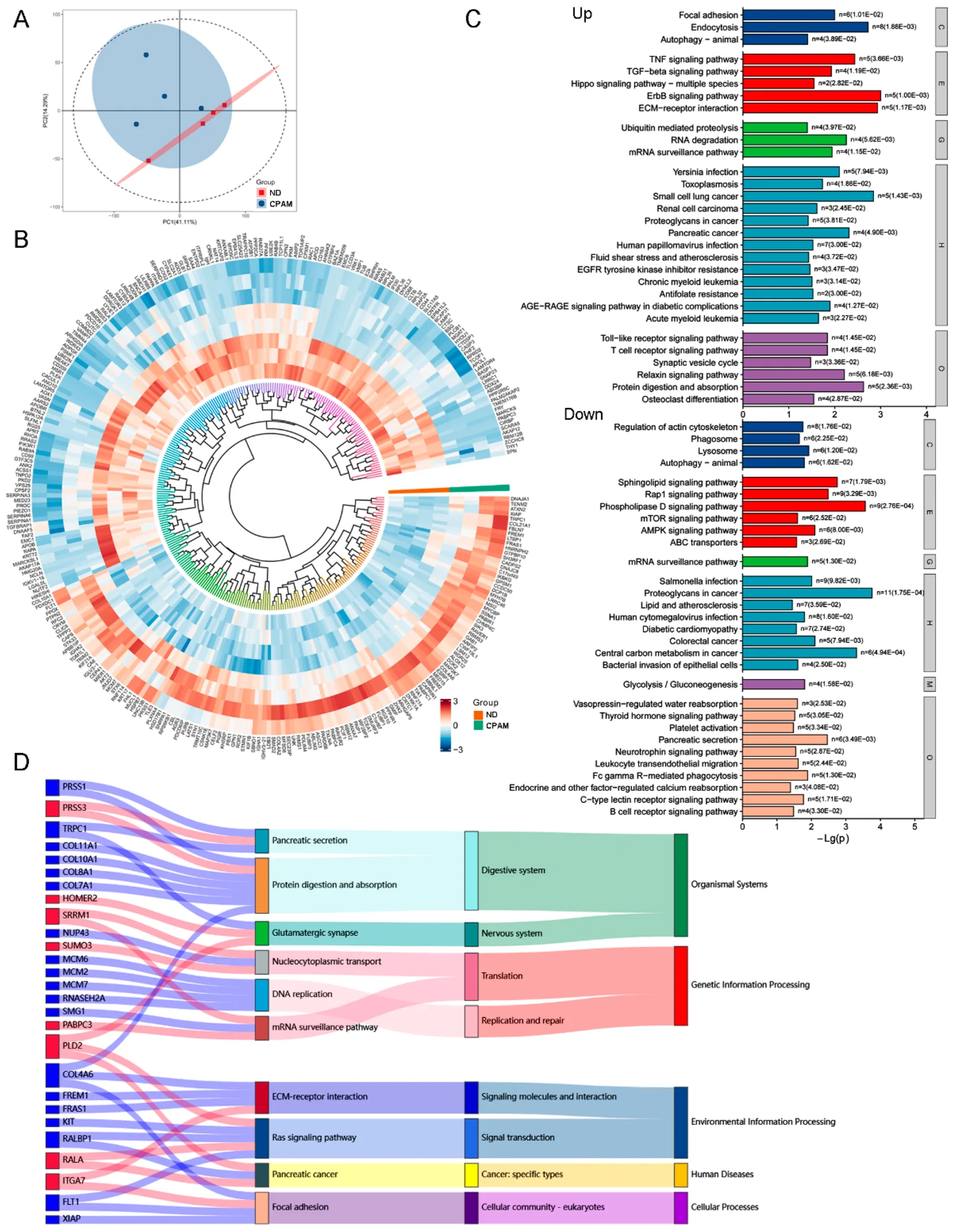

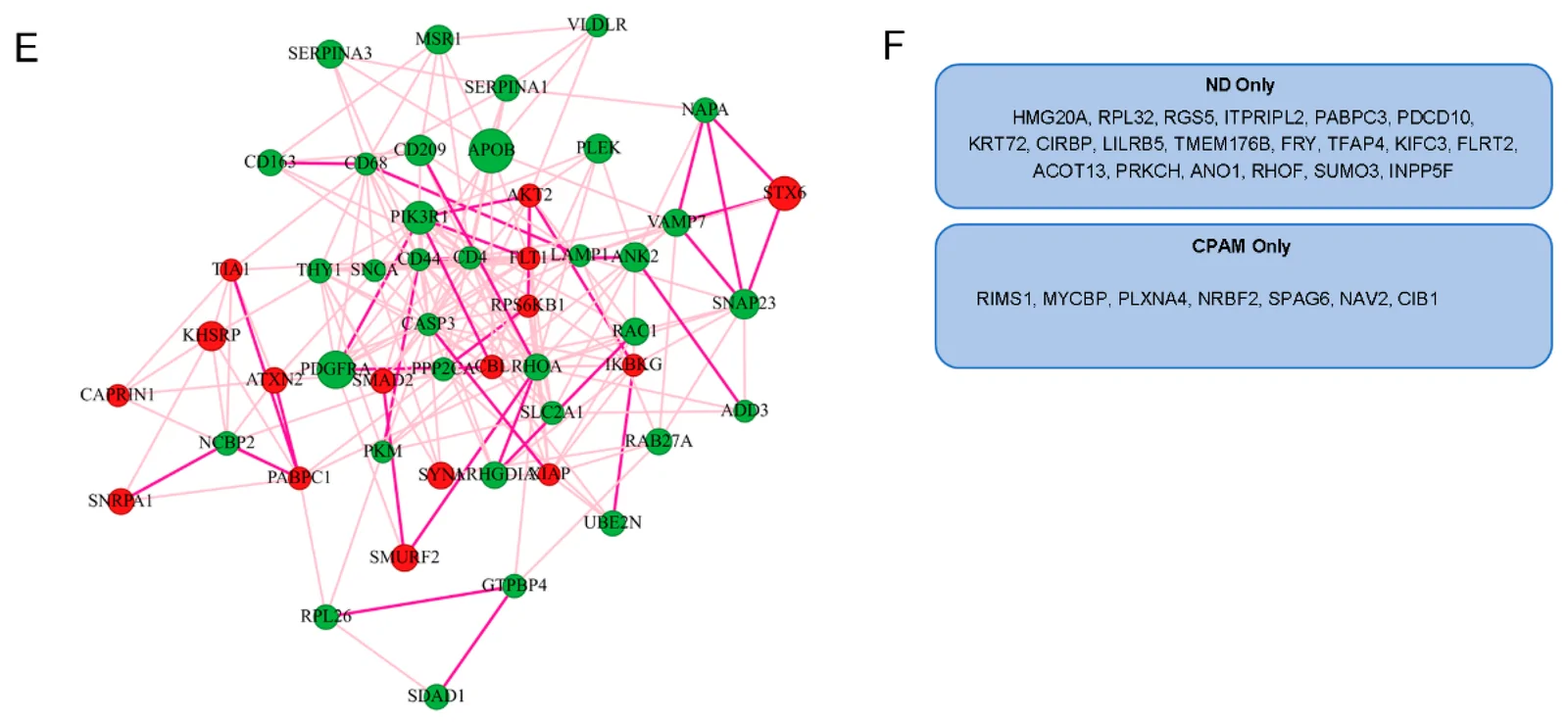
ND vs. CPAM lung ECM composition. (**A**) PCA plot demonstrating the similarity of the ECM protein composition within each respective decellularized sample. (**B**) Circle heatmap of differentially expressed proteins (DEPs) between ND- and CPAM-decellularized lung ECM. (**C**) KEGG pathway analysis of upregulated and downregulated pathways between ND- and CPAM-decellularized lung ECM. (**D**) Sankey diagram of the relationships between the top 10 signaling pathways and their DEPs. (**E**) PPI analysis of the networks of DEPs that collaborate to perform biological processes. (**F**) Differences in the levels of ECM proteins between groups of ND- and CPAM-decellularized lungs.

DIA proteomic analyses revealed a range of proteins in the decellularized lungs. Principal component analysis (PCA) demonstrated that samples derived from ND lungs presented remarkably similar proteomic compositions (Figure 2A). As displayed in the circle heatmap, 431 differentially expressed proteins (DEPs) were identified using differential expression analysis, of which 171 were upregulated and 260 were downregulated in CPAM lungs compared with those in ND decellularized lungs (Figure 2B). A complete list of all DEPs is depicted in Table S1. The KEGG pathway analysis revealed that the upregulated pathways included the “TGF-β signaling pathway”, “ECM-receptor interaction”, “mTOR signaling pathway”, “NOD-like signaling pathway”, “PI3K-Akt signaling pathway” and “Notch signaling pathway”, whereas the downregulated pathways included the “Sphingolipid signaling pathway”, “MAPK signaling pathway”, “phospholipase D signaling pathway” and “Hedgehog signaling pathway” in CPAM-decellularized lungs (Figure 2C and Figure S1). A Sankey diagram illustrates the relationships between the top 10 signaling pathways and their DEPs (Figure 2D). Through protein–protein interaction (PPI) analysis, the networks of DEPs that collaborate to perform biological processes were revealed (Figure 2E). Furthermore, among DEPs, CPAM specifically regulates synaptic membrane exocytosis protein 1 (RIMS1), plexin A4 (PLXNA4) and neuron navigator 2 (NAV2), which are critical for axon guidance and neuronal migration in the developing nervous system. On the other hand, ND-specific DEPs play important roles in various biological processes, such as cell signaling, metabolism, immune regulation and neurodevelopment (Figure 2F). The above results support findings from previous studies on CPAM and validate the reliability of the enrichment of decellularized ECM proteomics data.

### 3.3. ECM Composition of Decellularized Lungs from CPAM

**Figure 3:**
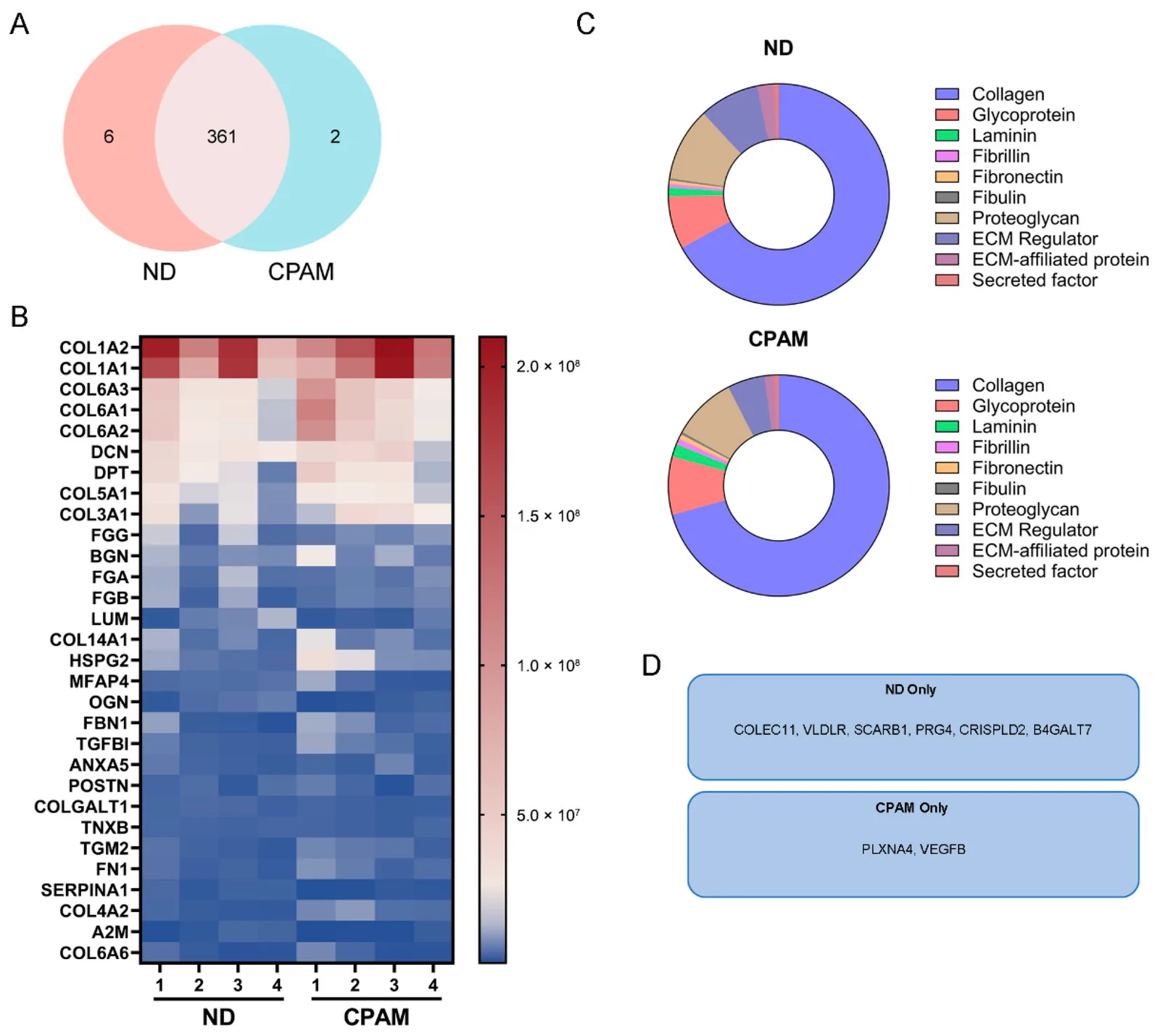
ND vs. CPAM lung basement membrane in terms of ECM composition. (**A**) Venn diagram of differences in the ECM composition of the basement membrane. (**B**) Heatmap of the top 30 normalized ECM proteins across all decellularized lung regions. (**C**) Ratio of the mean basement membrane composition of decellularized lungs. (**D**) Differences in the ECM composition of the basement membrane.

ECM proteins were then selected and classified into subgroups according to their collagens, ECM glycoproteins, ECM regulators, ECM-affiliated proteins, proteoglycans, or secreted factors. The most abundant proteins detected in ND lungs were basement membrane-associated proteins, including collagens (represented by type I and type VI), decorin (DCN), dermatopontin (DPT), fibrinogens, biglycan (BGN), lumican (LUM), and heparan sulfate proteoglycan-2/perlecan (HSPG2) (Figure 3B), which is different from characterizing the ECM composition of adult ND lungs. Analysis of the relative proportions and compositions of basement membrane-associated proteins revealed that, compared with ND lungs, CPAM lungs were enriched with more basement membrane-associated proteins, including collagens, laminins, fibronectin, fibrillin and fibulins, but fewer ECM regulators and ECM-affiliated proteins (Figure 3C). Among the ECM proteins, CPAM showed specific expression of PLXNA4 and vascular endothelial growth factor B (VEGFB), which play roles in angiogenesis and tumor metastasis. On the other hand, ND samples showed enriched expression of ECM proteins such as collectin 11 (COLEC11, *p* = 0.041), very low-density lipoprotein receptor (VLDLR, *p* = 0.042), scavenger receptor class B member 1 (SCARB1, *p* = 0.183), proteoglycan 4 (PRG4, *p* = 0.170), cysteine-rich secretory protein LCCL domain-containing 2 (CRISPLD2, *p* = 0.038) and β-1,4-galactosyltransferase 7 (B4GALT7, *p* = 0.047) (Figure 3D), which participate in biological processes such as the immune response, inflammation regulation, and tissue repair.

### 3.4. Differences Between ND and Diseased CPAM Lungs

**Figure 4:**
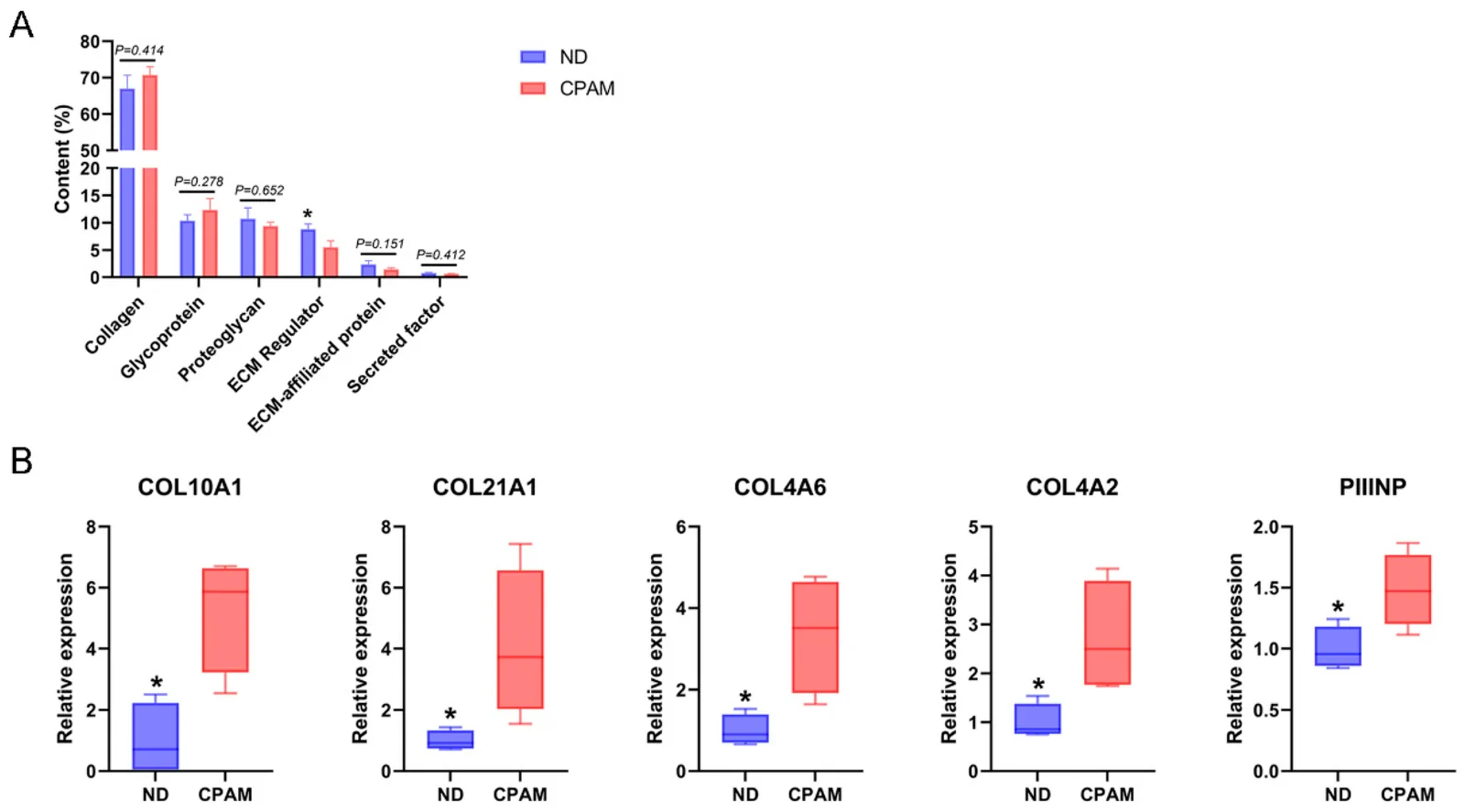
Proteomic analysis of collagens from ND- and CPAM-decellularized patient lungs. (**A**) Relative abundance (%) of ECM protein types in ND- and CPAM-decellularized lungs. (**B**) Comparison of normalized individual collagens in ND- and CPAM-decellularized lungs. The bars indicate the means ± SEMs. * *p* < 0.05 versus the ND group.

Analysis of the relative abundance of ECM protein types revealed an enrichment of collagens (66.98 ± 3.69% vs. 70.72 ± 2.26%, *p* = 0.414), glycoproteins (10.37 ± 1.12% vs. 12.33 ± 2.11%, *p* = 0.278), proteoglycans (10.72 ± 2.04% vs. 9.39 ± 0.73%, *p* = 0.652), ECM regulators (8.80 ± 0.98% vs. 5.48 ± 1.20%, *p* = 0.034), ECM-affiliated proteins (2.37 ± 0.67% vs. 1.46 ± 0.30%, *p* = 0.151) and secreted factors (0.765 ± 0.16% vs. 0.63 ± 0.06%, *p* = 0.412) in CPAM lungs and ND lungs respectively (Figure 4A). While overall collagens were not significantly increased in CPAM, subunits of COL4 (COL4A6, COL4A2), COL10A1, COL21A1 and the N-terminal propeptide of type III procollagen (PIIINP) in CPAM were significantly increased (Figure 4B). To validate the proteomic findings, we interrogated the public transcriptomic dataset GSE190620 (Figure S2).

**Figure 5:**
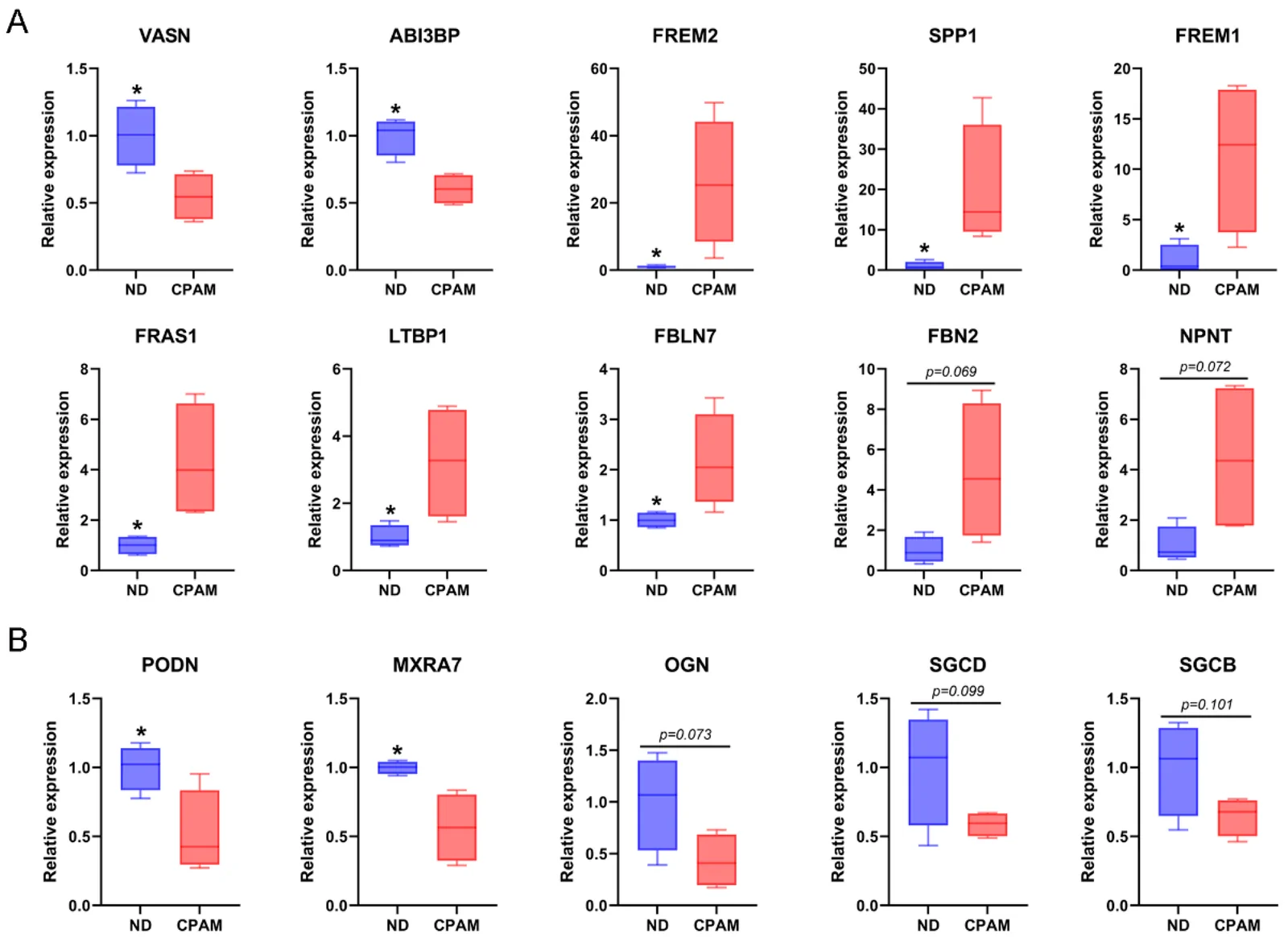
Proteomic analysis of glycoproteins and proteoglycans from ND- and CPAM-decellularized patient lungs. (**A**) Comparison of normalized individual glycoproteins in ND- and CPAM-decellularized lungs. (**B**) Comparison of normalized individual proteoglycans in ND- and CPAM-decellularized lungs. The bars indicate the means ± SEMs. * *p* < 0.05 versus the ND group.

With respect to the enrichment of total ECM glycoproteins in CPAM, several individual proteins, including ABI family member 3 binding protein (ABI3BP) and vasorin (VASN), were decreased, whereas the levels of the extracellular matrix organizing proteins FRAS1; FRAS1-related extracellular matrix proteins 1 and 2 (FREM1 and FREM2); osteopontin (SPP1); latent-transforming growth factor beta-binding protein 1 (LTBP1); and fibulin 7 (FBLN7) were increased, with a trending increase in fibrillin 2 (FBN2) and nephronectin (NPNT) (Figure 5A). Some of these glycoproteins can regulate the assembly and stability of the ECM, and their abnormal expression may be associated with the malformation of pulmonary airway structures (Figure 5A and Figure S3).

Among the enriched total ECM proteoglycans in CPAM, podocan (PODN) and matrix remodeling associated 7 (MXRA7) significantly decreased, with a trending decrease in mimecan (OGN) and β- and δ-sarcoglycan (SGCD and SGCB) (Figure 5B and Figure S4), which are reported to regulate ECM assembly, stability and remodeling and mediate the stability of the cytoskeleton and cellular behavior.

**Figure 6:**
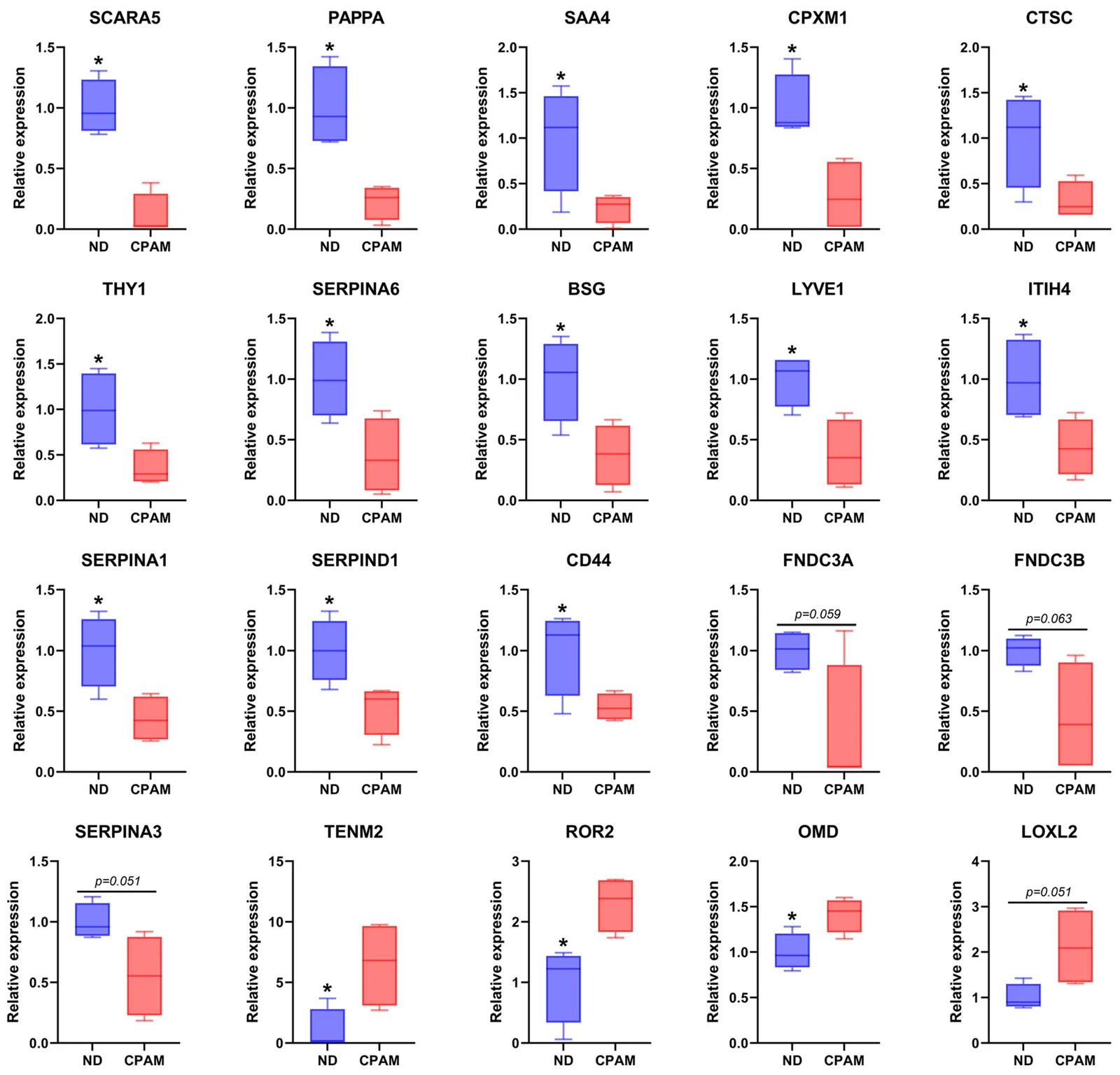
Proteomic analysis of ECM regulators from ND- and CPAM-decellularized patient lungs. The bars indicate the means ± SEMs. * *p* < 0.05 versus the ND group.

Notably, a decrease in the expression of ECM regulators—including scavenger receptor class A member 5 (SCARA5), pappalysin-1 (PAPPA), carboxypeptidase X1 (CPXM1), lymphatic vessel endothelial hyaluronic acid receptor 1 (LYVE1), α-1-antitrypsin (SERPINA1), basigin (BSG), corticosteroid-binding globulin (SERPINA6), heparin cofactor 2 (SERPIND1), THY1 membrane glycoprotein, interα-trypsin inhibitor heavy chain H4 (ITIH4), serum amyloid A-4 protein (SAA4), CD44 and cathepsin C (CTSC)—was observed in CPAM, whereas the tyrosine-protein kinase transmembrane receptor ROR2, osteomodulin (OMD), and teneurin-2 (TENM2) showed an increase in ECM regulators. A trending decrease in fibronectin type III domain-containing protein 3A and 3B (FNDC3A and FNDC3B) and α-1-antichymotrypsin (SERPINA3), and a trending increase in lysyl oxidase homolog 2 (LOXL2) were detected (Figure 6 and Figure S5).

**Figure 7:**
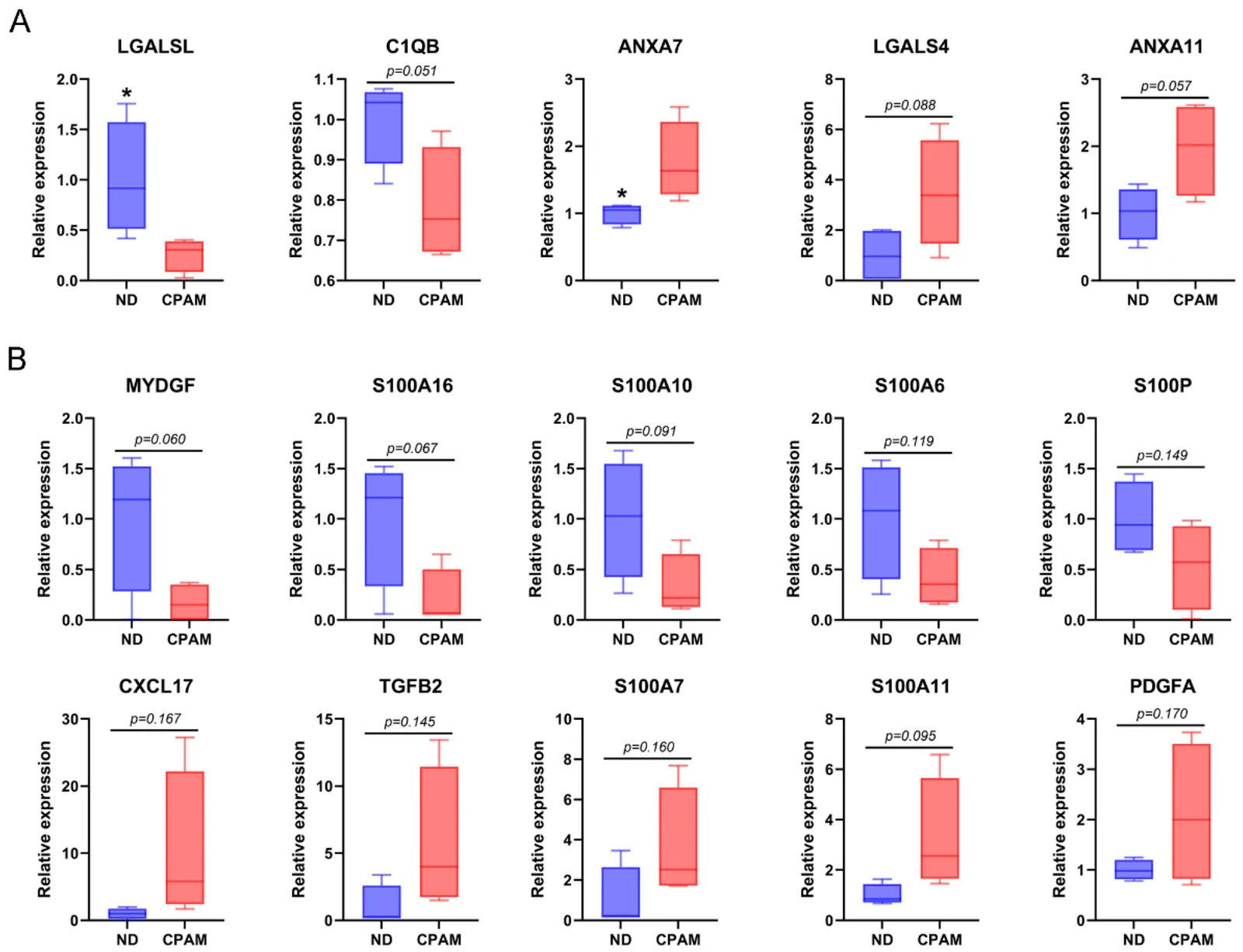
Proteomic analysis of ECM-affiliated proteins and secreted factors from ND- and CPAM-decellularized patient lungs. (**A**) Comparison of normalized individual ECM-affiliated proteins in ND- and CPAM-decellularized lungs. (**B**) Comparison of normalized individual secreted factors in ND- and CPAM-decellularized lungs. The bars indicate the means ± SEMs. * *p* < 0.05 versus the ND group.

Among the ECM-affiliated proteins, galectin-related protein (LGALSL) and complement C1q subcomponent subunit B (C1QB) were increased, whereas annexin A7 and A11 (ANXA7 and ANXA11) and galectin-4 (LGALS4) were decreased (Figure 7A and Figure S6). While the levels of no secreted factors significantly changed in CPAM, the levels of myeloid-derived growth factor (MYDGF) and several S100 protein subsets tended to decrease. The levels of C-X-C motif chemokine 17 (CXCL17), transforming growth factor β2 (TGFB2) and platelet-derived growth factor subunit A (PDGFA) showed a non-significant increasing trend (Figure 7B and Figure S7).

### 3.5. In Vitro Evaluation of the Lung Decellularized ECM Hydrogel

**Figure 8:**
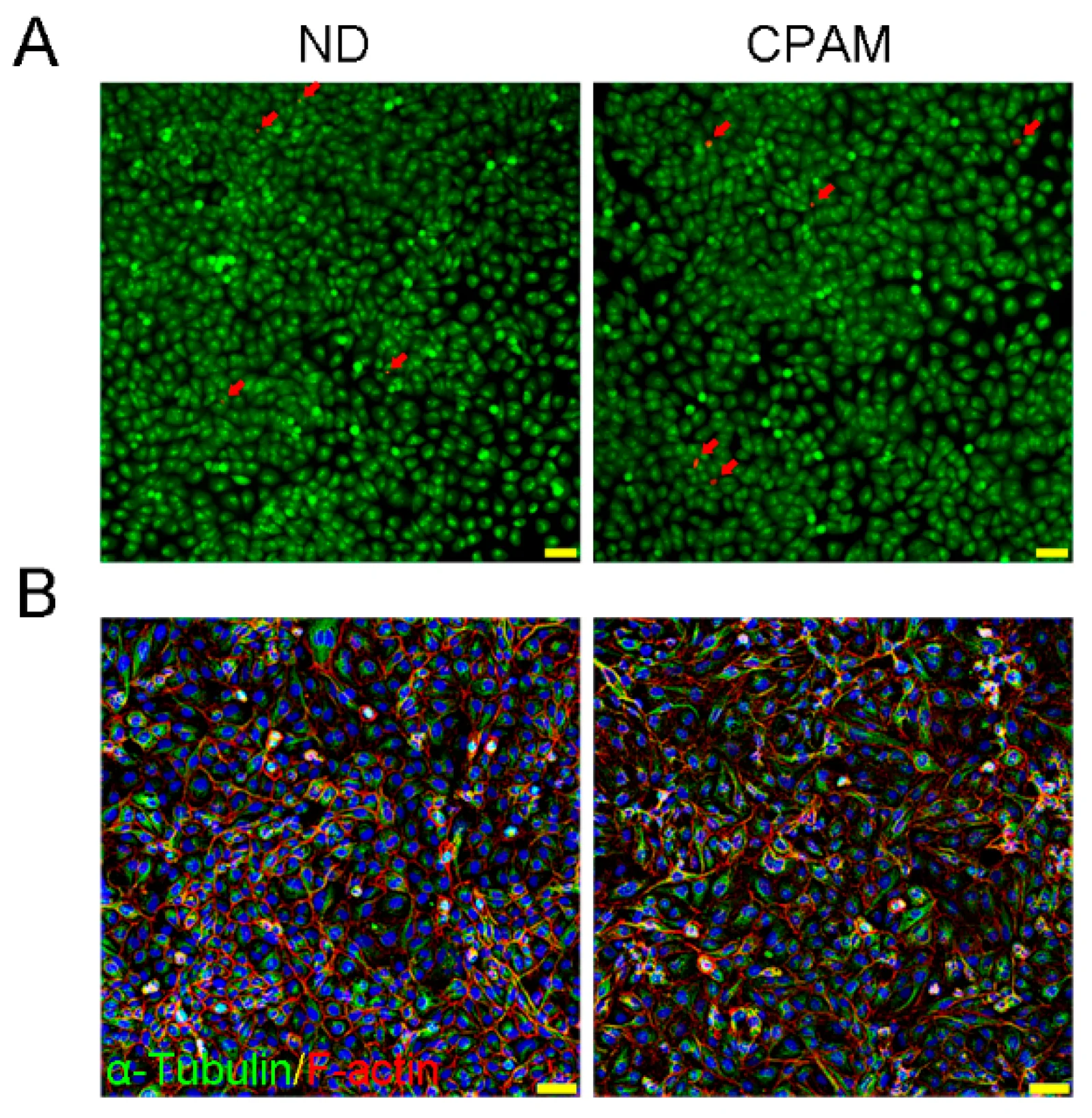
In vitro evaluation of the lung decellularized ECM hydrogel. (**A**) Live (calcein AM, green)/dead (propidium iodide, red) staining of HUVECs cultured on ND- and CPAM-decellularized ECM hydrogels. (**B**) Rhodamine phalloidin F-actin staining (red) and α-tubulin (green) staining of HUVECs cultured on ND- and CPAM-decellularized ECM hydrogels. Scale bar = 50 μm.

To evaluate the characteristics of the lung decellularized ECM, corresponding hydrogels were seeded with HUVECs as a proof-of-concept cellular model since they are highly sensitive to microenvironmental cues and widely used for initial assessment of biomaterial properties. Cell survival of HUVECs was higher on ND-decellularized ECM hydrogel than on CPAM-lung-decellularized ECM hydrogel, according to live/dead calcein AM/propidium iodide analysis (Figure 8A). The reason may be related to ECM composition, and mechanical stress in CPAM-affected regions can trigger endothelial injury. Furthermore, there were more adherent cells with the highest degree of expansion, distribution and connection on the ND lung decellularized ECM hydrogel (Figure 8B). Lung decellularized ECM hydrogel can be used as a cell culture material to determine pathological cellular behaviors, to evaluate mechanical properties and degradation, and to establish pathological modeling applications in vitro.

## 4. Discussion and Conclusions

ECM is increasingly recognized to play important roles in normal lung homeostasis and in lung disease pathogenesis [7]. Prior studies have largely relied on RNA-seq and single-cell RNA-seq technologies to characterize transcriptomic changes in CPAM patients [21,22,23,24]. IHC and mass spectrometry approaches were utilized to detect the cellular organization and tissue composition at the protein level [25,26]. Recent work has highlighted the presence of atypical mesenchyme in CPAM, suggesting that mesenchymal cell dysfunction may be a key driver of lesion formation [27]. Furthermore, a single-cell sequencing study has further refined our understanding of CPAM cellular heterogeneity. The researchers demonstrated that red blood cells, plasma cells, and mast cells are significantly increased in CPAM lesions, with CCL5 and NKG7 emerging as key differentially expressed genes involved in intercellular communication [28]. In this study, we used a decellularization method to first characterize the global ECM composition of cystic areas and their paired control areas from CPAM lungs. Our ECM-centric approach complements recent single-cell transcriptomic studies that have mapped the cellular landscape of CPAM. For instance, another study demonstrated that alveolar type 1 and type 2 cells, club cells, and basal cells are significantly increased in CCAM lesions, with mast cell–epithelial cell interactions potentially driving epithelial proliferation [29]. The ECM alterations we identified may represent the extracellular microenvironment that supports or arises from these aberrant cellular populations. Compared with ND areas derived from CPAM patients, cystic areas presented unique proteomic signatures. The primary collagens, glycoproteins, proteoglycans, and an array of ECM regulators displayed different ECM composition profiles. Pathway analysis highlighted that dysregulation of TGF-β, PI3K–AKT, and mTOR signaling was observed in CPAM, which may be associated with aberrant ECM–cell interactions in pathogenesis. Notably, CPAM exhibited elevated fibrillar collagens and disrupted ECM turnover, suggesting a maladaptive repair phenotype. These preliminary findings provide an initial comprehensive proteomic atlas of decellularized CPAM ECM alterations, offering exploratory insights into disease mechanisms and potential therapeutic targets. Given the small cohort size (*n* = 4), these results should be considered hypothesis-generating and warrant validation in larger independent cohorts. The proteomic results of decellularized lung ECM were consistent with RNA-seq data, while also revealing low-abundance ECM alterations that could not be detected by RNA-seq alone (Figures S2–S7). This study underscores the utility of decellularized ECM for modeling congenital lung disorders and informs future biomaterial-based interventions.

Specifically, CPAM cystic areas were enriched in collagens, including COL10A1, COL21A1, COL4 and PIIINP. However, their role in lung development, function, and pathology in CPAM remains poorly understood. COL4 is known to be present within the basement membrane of the lung, such as the alveolar–capillary basement membrane, and has been demonstrated to provide critical support for gas exchange. Recently, COL4 was shown to be disrupted in the epithelial-to-mesenchymal transition process and to impair the PI3K-AKT signaling pathway. COL10A1 possibly contributes to branching morphogenesis and vascular restructuring. As a fibril-associated collagen (FACIT family), COL21A1 may regulate the assembly of other collagens to influence lung elasticity. PIIINP modulates type III collagen synthesis and alveolarization. However, this is not entirely consistent with previous studies of CPAM. Several studies have shown a decrease in collagen at both the mRNA and protein levels in CPAM through RT–PCR and immunohistochemical staining, whereas our results indicate an increase in collagen. This discrepancy may be related to an increased turnover rate of collagen, where both its synthesis and degradation are elevated, particularly under the influence of recurrent pulmonary infections. The solubilization of collagen fragments in cystic regions of the CPAM lungs increased their detection and enrichment. As observed in previous proteomic studies of decellularized human lungs, the primary subunits of COL6, such as COL6A1, COL6A2, and COL6A3, were consistently among the most abundant ECM proteins identified across all samples independent of the lung condition [26]. In our study, we found that the most abundant collagens were subunits of COL1, i.e., COL1A2 and COL1A1, in the ND areas of patients. COL1 mainly provides structural support and tensile resistance, forming coarse fibrils that endow the lung tissue with mechanical robustness against deformation. It also collaborates with elastin fibers to preserve the pulmonary elastic recoil force during respiration. Thus, dysregulated type I/III collagen ratios in CPAM can also affect the mechanical properties of cystic regions, including tissue stiffness, elasticity, and structural integrity (Figure S8). A lower abundance of COL6 in the lungs might destabilize the alveolar microstructure. These findings indicate the importance of further evaluation of the role of collagens, such as COL4, in lung physiology and disease progression. In addition, laminins, a key subset of basement membrane glycoproteins, were not enriched in the top 30 proteins, indicating their developmental stage-specific expression patterns.

The proportion of ECM glycoproteins within the total ECM composition was also greater in CPAM than in ND. As ECM glycoproteins provide a vast array of biochemical cues, through attaching glycosaminoglycans and directly binding to cells, alterations in ECM glycoproteins under disease conditions likely have a significant impact on cell behavior and disease progression [19]. Within the ECM glycoprotein category, we demonstrated an increase in FRAS, FREM1 and FREM2 in CPAM, similar to previous studies demonstrating that recessive mutations in FRAS1, FREM2, and GRIP1 cause Fraser syndrome, which is characterized by cognitive impairments, cryptophthalmos, syndactyly, genital and renal anomalies and a range of other structural defects, including CDH, lung lobulation defects and anal anomalies [30]. In an early study, Beck et al. demonstrated that Frem1-deficient mice exhibit lung lobulation defects, a phenotype modulated by genetic interaction with Gata4, which is essential for normal pulmonary lobar development. In their study, the addition of a Gata4-null allele significantly increased the prevalence of lung lobulation defects in Frem1-mutant mice, suggesting that variations in GATA4 expression can modulate the penetrance of FREM1-related phenotypes. This genetic evidence supports the notion that the FRAS/FREM protein family is critical for proper lung morphogenesis, and our observation of their upregulation in CPAM may represent a compensatory or dysregulated repair response in the lesional microenvironment [30].

Rho-GTPases such as RHO, RAC1, and CDC42 are involved in the regulation of the cytoskeleton, cell growth, and the cell cycle. RhoA and Rac1 have been proven to regulate the wound closure of bronchial epithelial cells [31]. The expression of RAC1, RHO, ROCK2 and CDC42 was lower in cystic areas in CPAM than in those in ND (Figure S9). A recent study revealed increased levels of the transcription factor Krüppel-like factor 5 (KLF5) in epithelial cells lining dilated tubules in the lungs of patients with CPAM. KLF5 can regulate actin asymmetry and induce epithelial cell shape changes by balancing RHOA and CDC42 GTPase activity via RICH2 [32].

In GSEA, multiple signaling pathways were demonstrated to contribute to CPAM. We found that ECM proteins were predominantly enriched in the PI3K-AKT-mTOR signaling pathway, indicating an important role for genes in this pathway in the pathogenesis of CPAM, which has been verified in previous studies [21,24,33]. Notably, a recent integrative analysis of bulk and single-cell RNA sequencing specifically focused on type II CPAM confirmed the critical involvement of the PI3K-AKT signaling pathway, along with GPCR ligand binding and the complement cascade, in this subtype [24]. The convergence of transcriptomic and proteomic evidence across multiple studies and CPAM subtypes strengthens the hypothesis that PI3K-AKT dysregulation is a core feature of CPAM pathogenesis, potentially driven by or reflected in the altered ECM microenvironment. Abnormal activation of the TGF-β signaling pathway has also been recognized in recent studies [23,34], and this finding was confirmed by defective TGF-β signaling in the lung mesenchyme and subsequent abnormal airway branching morphogenesis and congenital airway cystic lesions in a mouse model [35]. A compartment-specific transcriptomic analysis of a CPAM epithelium demonstrated that TGFB2 and TGFBR1 were significantly upregulated in the cystic epithelium compared to control areas, with immunohistochemistry confirming increased protein levels [36]. Our ECM-focused proteomic findings extend this observation by showing that TGF-β pathway enrichment is not limited to the epithelial compartment but is also reflected in the ECM composition of the lesional microenvironment. In addition, we also reported increased resistance to EGFR tyrosine kinase inhibitors. On the other hand, the enriched downregulated phospholipase D, Rap1, AMPK and VEGF signaling pathways in this study may explain the pathologic features and serve as novel targets for CPAM. Inhibition of the phospholipase D signaling pathway may impair surfactant secretion, weaken bacterial clearance, and, together with reduced VEGF signaling, aggravate vascular leakage and impair endothelial repair. Rap1 inhibition disrupts VE-cadherin function, increasing vascular permeability and leading to endothelial barrier dysfunction. Rap1 deficiency may also promote smooth muscle cell proliferation and increase the risk of developing hypoxia-induced pulmonary arterial hypertension. Impaired integrin-mediated lymphocyte adhesion may modify pulmonary inflammation. AMPK downregulation increases oxidative stress, amplifying the proinflammatory effects and pulmonary smooth muscle cell proliferation. The combined downregulation synergistically disrupts pulmonary vascular function and the inflammatory balance and impairs repair, thus accelerating the progression of disease and complications. Beyond transcriptional and proteomic changes, epigenetic mechanisms may also contribute to CPAM pathogenesis. Patrizi et al. performed whole-genome methylation profiling of congenital lung malformations and identified differentially methylated regions in genes involved in cell proliferation and embryonic development, some of which have been implicated in lung tumors [37]. This raises the intriguing possibility that the ECM alterations we observed could be driven or sustained by epigenetic modifications, and that the ECM itself may influence the epigenetic landscape of resident cells through mechanotransduction and biochemical signaling.

Beyond the structural and biochemical cues provided by the ECM, intercellular communication via extracellular vesicles (EVs) represents a complementary mechanism that may modulate ECM remodeling and cellular responses in CPAM [38,39]. EVs carry proteins, lipids, and nucleic acids between cells and have been shown to regulate tissue repair, cell differentiation, and disease pathogenesis in various pulmonary conditions. Given that ECM composition can influence EV uptake and cargo delivery, and that EVs can in turn deliver ECM-modifying enzymes (e.g., MMPs) to recipient cells, a bidirectional crosstalk between ECM and EV-mediated signaling may exist [39,40]. Future studies exploring the EV cargo and functional effects in CPAM-derived ECM microenvironments could further elucidate the complex intercellular networks that contribute to disease pathogenesis and identify novel therapeutic targets. Our ECM hydrogel platform could serve as a valuable tool to investigate how disease-specific ECM modulates EV production and function.

In summary, the methodology presented in this study provides a novel and simple method for identifying and characterizing human lung ECM proteins via advanced proteomics. We demonstrated that CPAM-specific cystic areas contain specific ECM signatures at both the structural and biochemical ECM protein levels. These proteomic ECM differences between ND and CPAM lungs could pave the way for a proteomic baseline and provide novel biotherapeutic targets for future studies. Furthermore, disease-specific ECM derivatives, such as scaffolds and hydrogels, are amenable to future biomaterial studies for ex vivo modeling applications to determine key cell-ECM interactions that contribute to pathological cellular behaviors [41,42,43].

## Figures and Tables

**Table 1:** Patient information.

Characteristics	DZX	ZKZ	HJR	ZSC	Mean ± SD
Gender	Female	Female	Male	Male	
Age (months)	24	11	7	24	16.50 ± 7.63
Body weight (kg)	12	8	10	14	11.00 ± 2.24
WBC counts (×10^9^/L)	15.6	10.9	9.09	7.48	10.77 ± 3.04
RBC counts (×10^12^/L)	5.02	4.19	3.78	4.81	4.45 ± 0.49
PLT counts (×10^9^/L)	342	438	219	297	324 ± 79.17
NEUT counts (×10^9^/L)	2.54	2.91	2.19	3.74	2.85 ± 0.58
LYM counts (×10^9^/L)	11.97	6.5	5.62	3.03	6.78 ± 3.26
MNC counts (×10^9^/L)	0.74	0.75	0.73	0.5	0.68 ± 0.10
Lesion site	Left lower	Left upper	Left lower	Right lower	
Size (cm)	4.5 × 4.0	1.9 × 1.8	4.3 × 4.0	3.5 × 2.0	

WBC, white blood cell; RBC, red blood cell; PLT, platelet; NEUT, neutrophil; LYM, lymphocyte; MNC, monocyte. All patients included in this analysis had no evidence of active infection at the time of surgery, as confirmed by clinical assessment and laboratory findings. The lesion size was determined by CT imaging.

## Data Availability

The original contributions presented in the study are included in the article/Supplementary Material; further inquiries can be directed to the corresponding author.

## Supplementary Materials

The following supporting information can be downloaded at https://www.mdpi.com/article/10.3390/jcm15176742/s1: Figure S1. The GSEA analysis of selected dysregulated pathways in CPAM decellularized lungs compared to ND decellularized lungs. Figure S2. Analysis of collagens from ND- and CPAM patient lungs in dataset GSE190620. Figure S3. Analysis of glycoproteins from ND- and CPAM patient lungs in dataset GSE190620. Figure S4. Analysis of proteoglycans from ND- and CPAM patient lungs in dataset GSE190620. Figure S5. Analysis of ECM regulators from ND- and CPAM patient lungs in dataset GSE190620. Figure S6. Analysis of ECM-affiliated proteins from ND- and CPAM patient lungs in dataset GSE190620. Figure S7. Analysis of secreted factors from ND- and CPAM patient lungs in dataset GSE190620. Figure S8. The ratios of type I/III collagen were lower in cystic areas in CPAM than in those in ND. Figure S9. The ratios of RAC1/RHOA were lower in cystic areas in CPAM than in those in ND; Table S1: A complete list of all differentially expressed proteins (DEPs) in decellularized ND and CPAM lungs.

## Abbreviations

CPAM	Congenital pulmonary airway malformation
ECM	Extracellular matrix
DIA	Data-independent acquisition
ND	Non-diseased
DEPs	Differentially expressed proteins
CCAM	Congenital cystic adenomatoid malformation
CT	Computed tomography
COPD	Chronic obstructive pulmonary disease
IPF	Idiopathic pulmonary fibrosis
COL1	Type I collagen
COL3	Type III collagen
WBC	White blood cell
RBC	Red blood cell
PLT	Platelet
NEUT	Neutrophil
LYM	Lymphocyte
MNC	Monocyte
OCT	Optimal cutting temperature
PBS	Phosphate-buffered saline
ddH_2_O	Double-distilled water
SLES	Sodium lauryl ether sulfate
H&E	Hematoxylin and eosin
SEM	Scanning electron microscopy
SDS	Sodium dodecyl sulfate
DTT	Dithiothreitol
IAA	Iodoacetamide
KEGG	Kyoto Encyclopedia of Genes and Genomes
GO	Gene Ontology
BP	Biological process
MF	Molecular function
CC	Cellular component
PPI	Protein–protein interaction
GEO	Gene Expression Omnibus
HUVECs	Human umbilical vein endothelial cells
SEMs	Standard errors of the means
PCA	Principal component analysis
RIMS1	Regulates synaptic membrane exocytosis protein 1
PLXNA4	Plexin A4
NAV2	Neuron navigator 2
DPT	Dermatopontin
BGN	Biglycan
LUM	Lumican
HSPG2	Heparan sulfate proteoglycan-2/perlecan
VEGFB	Vascular endothelial growth factor B
COLEC11	Collectin 11
VLDLR	Very low-density lipoprotein receptor
SCARB1	Scavenger receptor class B member 1
PRG4	Proteoglycan 4
CRISPLD2	Cysteine-rich secretory protein LCCL domain-containing 2
B4GALT7	β-1,4-galactosyltransferase 7
PIIINP	N-terminal propeptide of type III procollagen
ABI3BP	ABI family member 3 binding protein
VASN	Vasorin
LTBP1	Latent-transforming growth factor beta-binding protein 1
FBLN7	Fibulin 7
FBN2	Fibrillin 2
NPNT	Nephronectin
PODN	Podocan
MXRA7	Matrix remodeling associated 7
SCARA5	Scavenger receptor class A member 5
PAPPA	Pappalysin-1
CPXM1	Carboxypeptidase X1
LYVE1	Lymphatic vessel endothelial hyaluronic acid receptor 1
SERPINA1	α-1-antitrypsin
BSG	Basigin
SERPINA6	Corticosteroid-binding globulin
SERPIND1	Heparin cofactor 2
ITIH4	Interα-trypsin inhibitor heavy chain H4
SAA4	Serum amyloid A-4 protein
CTSC	Cathepsin C
OMD	Osteomodulin
TENM2	Teneurin-2
LOXL2	Lysyl oxidase homolog 2
LGALSL	Galectin-related protein
C1QB	Complement C1q subcomponent subunit B
ANXA7	Annexin A7
ANXA11	Annexin A11
CXCL17	C-X-C motif chemokine 17
TGFB2	Transforming growth factor β2
PDGFA	Platelet-derived growth factor subunit A
KLF5	Krüppel-like factor 5

## References

[1] Wong K.K.Y., Flake A.W., Tibboel D., Rottier R.J., Tam P.K.H. (2018). Congenital pulmonary airway malformation: Advances and controversies. Lancet Child Adolesc. Health.

[2] Doktor F., Antounians L., Lacher M., Zani A. (2022). Congenital lung malformations: Dysregulated lung developmental processes and altered signaling pathways. Semin. Pediatr. Surg..

[3] Xu W., Gao Y., Li W., Chen Z., Li Q., Li R., Liu H., Dai L. (2022). A national descriptive epidemiologic analysis on congenital pulmonary airway malformation in China, 2010–2019. Pediatr. Pulmonol..

[4] Danopoulos S., Alonso I., Thornton M.E., Grubbs B.H., Bellusci S., Warburton D., Al Alam D. (2018). Human lung branching morphogenesis is orchestrated by the spatiotemporal distribution of ACTA2, SOX2, and SOX9. Am. J. Physiol. Lung Cell Mol. Physiol..

[5] Swarr D.T., Peranteau W.H., Pogoriler J., Frank D.B., Adzick N.S., Hedrick H.L., Morley M., Zhou S., Morrisey E.E. (2018). Novel Molecular and Phenotypic Insights into Congenital Lung Malformations. Am. J. Respir. Crit. Care Med..

[6] Pederiva F., Rothenberg S.S., Hall N., Ijsselstijn H., Wong K.K.Y., von der Thüsen J., Ciet P., Achiron R., Pio d’Adamo A., Schnater J.M. (2023). Congenital lung malformations. Nat. Rev. Dis. Primers.

[7] Zhou Y., Horowitz J.C., Naba A., Ambalavanan N., Atabai K., Balestrini J., Bitterman P.B., Corley R.A., Ding B.-S., Engler A.J. (2018). Extracellular matrix in lung development, homeostasis and disease. Matrix Biol..

[8] Burgess J.K., Weiss D.J., Westergren-Thorsson G., Wigen J., Dean C.H., Mumby S., Bush A., Adcock I.M. (2024). Extracellular Matrix as a Driver of Chronic Lung Diseases. Am. J. Respir. Cell Mol. Biol..

[9] Burgess J.K., Harmsen M.C. (2022). Chronic lung diseases: Entangled in extracellular matrix. Eur. Respir. Rev..

[10] Naba A. (2024). Mechanisms of assembly and remodelling of the extracellular matrix. Nat. Rev. Mol. Cell Biol..

[11] Zhang H., Tsui C.K., Garcia G., Joe L.K., Wu H., Maruichi A., Fan W., Pandovski S., Yoon P.H., Webster B.M. (2024). The extracellular matrix integrates mitochondrial homeostasis. Cell.

[12] Kroemer G., Maier A.B., Cuervo A.M., Gladyshev V.N., Ferrucci L., Gorbunova V., Kennedy B.K., Rando T.A., Seluanov A., Sierra F. (2025). From geroscience to precision geromedicine: Understanding and managing aging. Cell.

[13] Statzer C., Park J.Y.C., Ewald C.Y. (2023). Extracellular Matrix Dynamics as an Emerging yet Understudied Hallmark of Aging and Longevity. Aging Dis..

[14] Burgstaller G., Oehrle B., Gerckens M., White E.S., Schiller H.B., Eickelberg O. (2017). The instructive extracellular matrix of the lung: Basic composition and alterations in chronic lung disease. Eur. Respir. J..

[15] Karakioulaki M., Papakonstantinou E., Stolz D. (2020). Extracellular matrix remodelling in COPD. Eur. Respir. Rev..

[16] Herrera J., Henke C.A., Bitterman P.B. (2018). Extracellular matrix as a driver of progressive fibrosis. J. Clin. Investig..

[17] Uhl F.E., Zhang F., Pouliot R.A., Uriarte J.J., Enes S.R., Han X., Ouyang Y., Xia K., Westergren-Thorsson G., Malmström A. (2020). Functional role of glycosaminoglycans in decellularized lung extracellular matrix. Acta Biomater..

[18] Hoffman E.T., Uhl F.E., Asarian L., Deng B., Becker C., Uriarte J.J., Downs I., Young B., Weiss D.J. (2022). Regional and disease specific human lung extracellular matrix composition. Biomaterials.

[19] HoHoffman E., Song Y., Zhang F., Asarian L., Downs I., Young B., Han X., Ouyang Y., Xia K., Linhardt R.J. (2023). Regional and disease-specific glycosaminoglycan composition and function in decellularized human lung extracellular matrix. Acta Biomater..

[20] Li Y., Wu Q., Li L., Chen F., Bao J., Li W. (2021). Decellularization of porcine whole lung to obtain a clinical-scale bioengineered scaffold. J. Biomed. Mater. Res. Part A.

[21] Yang W., Zhao P., Cao P., Miao C., Ji X., Gao Y., Li P., Cheng J. (2022). Global interpretation of novel alternative splicing events in human congenital pulmonary airway malformations: A pilot study. J. Cell Biochem..

[22] Tan Z., Li F., Chen Q., Chen H., Xue Z., Zhang J., Gao Y., Liang L., Huang T., Zhang S. (2023). Integrated bulk and single-cell RNA-sequencing reveals SPOCK2 as a novel biomarker gene in the development of congenital pulmonary airway malformation. Respir. Res..

[23] Zhang G., Lou L., Shen L., Zeng H., Cai C., Wu R., Liu D. (2024). The underlying molecular mechanism of ciliated epithelium dysfunction and TGF-β signaling in children with congenital pulmonary airway malformations. Sci. Rep..

[24] Li F., Tan Z., Chen H., Gao Y., Xia J., Huang T., Liang L., Zhang J., Zhang X., Shi X. (2024). Integrative analysis of bulk and single-cell RNA sequencing reveals the gene expression profile and the critical signaling pathways of type II CPAM. Cell Biosci..

[25] Jiang Y., Luo Y., Tang Y., Moats R., Warburton D., Zhou S., Lou J., Pryhuber G.S., Shi W., Wang L.L. (2019). Alteration of cystic airway mesenchyme in congenital pulmonary airway malformation. Sci. Rep..

[26] Barazzone-Argiroffo C., Maillard J.L., Vidal I., Bochaton-Piallat M.L., Blaskovic S., Donati Y., Wildhaber B.E., Rougemont A.-L., Delacourt C., Ruchonnet-Métrailler I. (2019). New insights on congenital pulmonary airways malformations revealed by proteomic analyses. Orphanet J. Rare Dis..

[27] Azar P., Avila Y., Hiltbrunner A., Delacourt C., Rougemont A.L., Vidal I., Bochaton-Piallat M.L., Ruchonnet-Metrailler I. (2025). Atypical mesenchyme in congenital pulmonary airways malformation: A promising new focus. Respir. Res..

[28] Huang J., Chen Q., Hong S., Hong J., Cao H. (2025). Single-cell sequencing reveals cellular differences and potential mechanisms in congenital pulmonary airway malformation. Front. Med..

[29] Zhang S., Ye C., Xiao J., Yang J., Zhu C., Xiao Y., Ye M., Chen Q. (2020). Single-cell transcriptome profiling reveals the mechanism of abnormal proliferation of epithelial cells in congenital cystic adenomatoid malformation. Exp. Cell Res..

[30] Beck T.F., Shchelochkov O.A., Yu Z., Kim B.J., Hernández-García A., Zaveri H.P., Bishop C., Overbeek P.A., Stockton D.W., Justice M.J. (2013). Novel frem1-related mouse phenotypes and evidence of genetic interactions with gata4 and slit3. PLoS ONE.

[31] Desai L.P., Aryal A.M., Ceacareanu B., Hassid A., Waters C.M. (2004). RhoA and Rac1 are both required for efficient wound closure of airway epithelial cells. Am. J. Physiol. Lung Cell Mol. Physiol..

[32] Li Q., Liao Y., Zeng J., Hu S., Li C., Whitsett J.A., Zheng Y., Luo F., Xu C., He T. (2024). KLF5 Shapes Developing Respiratory Tubules by Inhibiting Actin Asymmetry in Epithelial Cells. Am. J. Respir. Cell Mol. Biol..

[33] Zeng J., Liu W., Liang J., Peng J., Wang F., Tang J., Yang Q., Zhuang L., Huang D., Li L. (2021). Analysis of miRNA Profiles and the Regulatory Network in Congenital Pulmonary Airway Malformations. Front. Pediatr..

[34] Zhang G., Cai C., Li X., Lou L., Zhou B., Zeng H., Yan X., Liu D., Yu G. (2022). Application of second-generation sequencing in congenital pulmonary airway malformations. Sci. Rep..

[35] Miao Q., Chen H., Luo Y., Chiu J., Chu L., Thornton M.E., Grubbs B.H., Kolb M., Lou J., Shi W. (2021). Abrogation of mesenchyme-specific TGF-β signaling results in lung malformation with prenatal pulmonary cysts in mice. Am. J. Physiol.-Lung Cell. Mol. Physiol..

[36] Lezmi G., Vibhushan S., Bevilaqua C., Crapart N., Cagnard N., Khen-Dunlop N., Boyle-Freyssaut C., Hadchouel A., Delacourt C. (2020). Congenital cystic adenomatoid malformations of the lung: An epithelial transcriptomic approach. Respir. Res..

[37] Patrizi S., Pederiva F., D’aDamo A.P. (2021). Whole-Genome Methylation Study of Congenital Lung Malformations in Children. Front. Oncol..

[38] Debnath K., Heras K.L., Rivera A., Lenzini S., Shin J.W. (2023). Extracellular vesicle-matrix interactions. Nat. Rev. Mater..

[39] Kamra K., Lalam S.K., Srivastava S., Arumugam M.K., Boopathy L.K., Takkar S., Xia Z., Subramanian S.P. (2026). Multifaceted Role of Extracellular Vesicles: Intercellular Messengers to Therapeutic Applications: A Narrative Review. Health Sci. Rep..

[40] Wu Y., Toldo N., Fabbri M. (2026). The interplay between the extracellular matrix and extracellular vesicle-associated microRNAs. Cell Commun. Signal.

[41] Zhu X., Li Y., Yang Y., He Y., Gao M., Peng W., Wu Q., Zhang G., Zhou Y., Chen F. (2022). Ordered micropattern arrays fabricated by lung-derived dECM hydrogels for chemotherapeutic drug screening. Mater. Today Bio.

[42] Zhu X., Yang Y., Mao S., Liu Q., Li Y., Yang Y., Gao M., Bao J., Li W., Li Y. (2024). Lung dECM matrikine-based hydrogel reverses bleomycin-induced pulmonary fibrosis by suppressing M2 macrophage polarization. Biofabrication.

[43] Zhu X., Mao S., Yang Y., Liu X., Liu Q., Zhang N., Yang Y., Li Y., Gao M., Bao J. (2025). Biomimetic Topological Micropattern Arrays Regulate the Heterogeneity of Cellular Fates in Lung Fibroblasts between Fibrosis and Invasion. ACS Nano.

