# Supplementary material for: Composition and Function of Decellularized Human Lung Extracellular Matrix from Congenital Pulmonary Airway Malformation
Source: J Clin Med. 2026 Aug 30;15(17):6742. doi: 10.3390/jcm15176742 (PMC13566063; doi:10.3390/jcm15176742)
Supplement: Supplementary file 1 [file jcm-15-06742-s001.zip › Figure S1-S9.pdf]

## Supplementary figures

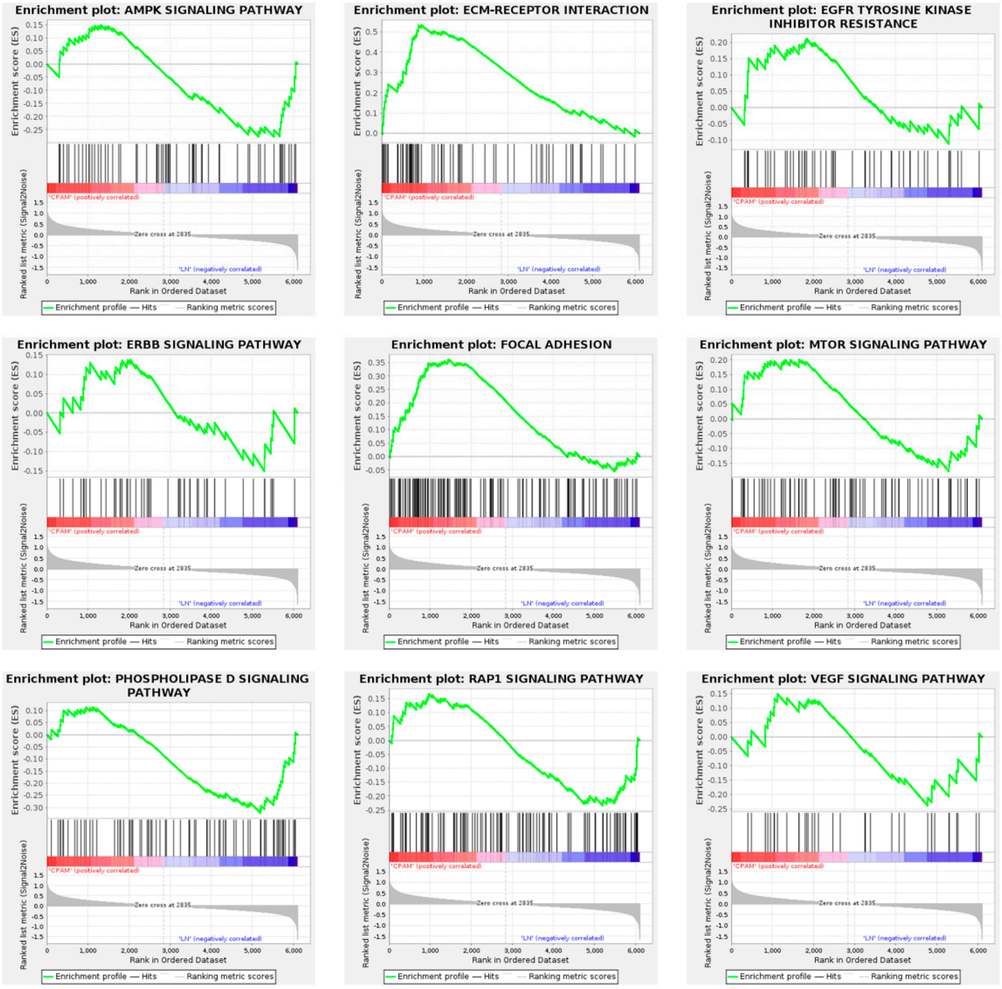


**Figure S1.** The GSEA analysis of selected dysregulated pathways in CPAM decellularized lungs compared to ND decellularized lungs.

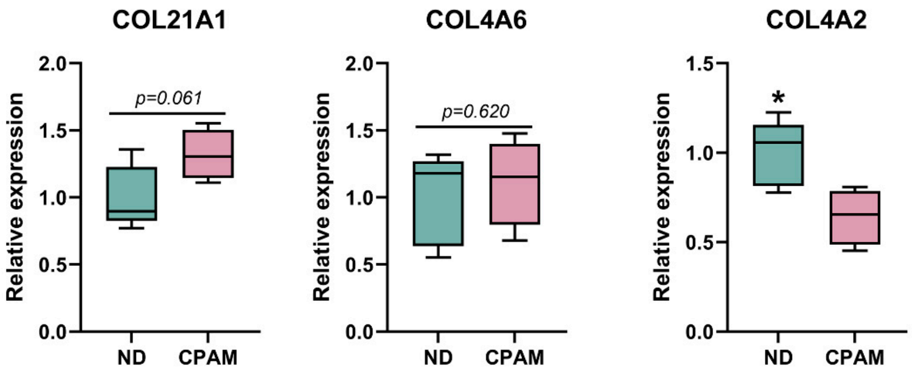


**Figure S2.** Analysis of collagens from ND- and CPAM patient lungs in dataset GSE190620.

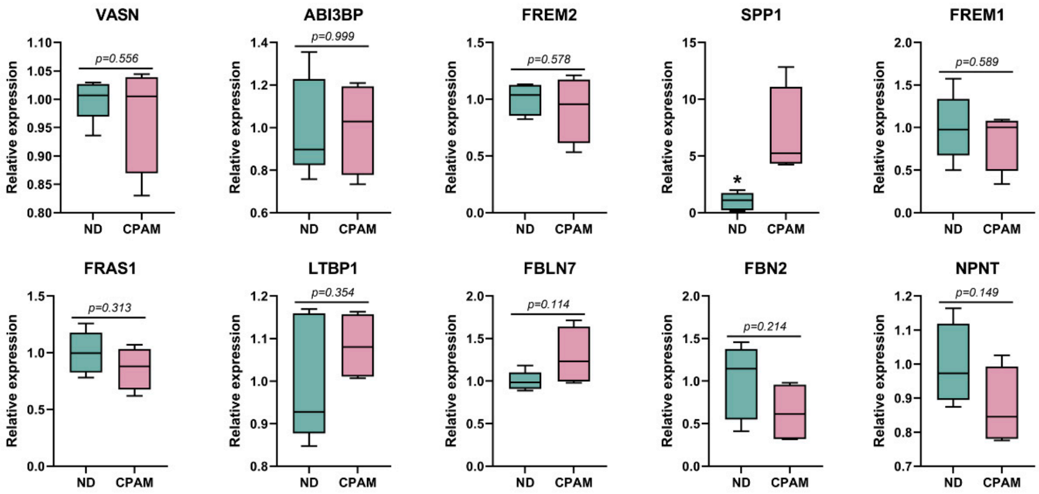


**Figure S3.** Analysis of glycoproteins from ND- and CPAM patient lungs in dataset GSE190620.

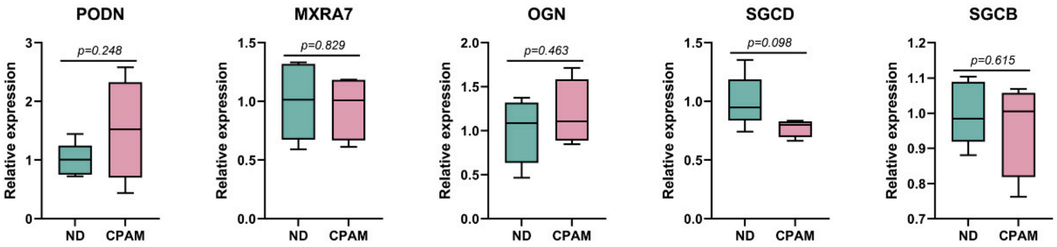


**Figure S4.** Analysis of proteoglycans from ND- and CPAM patient lungs in dataset GSE190620.

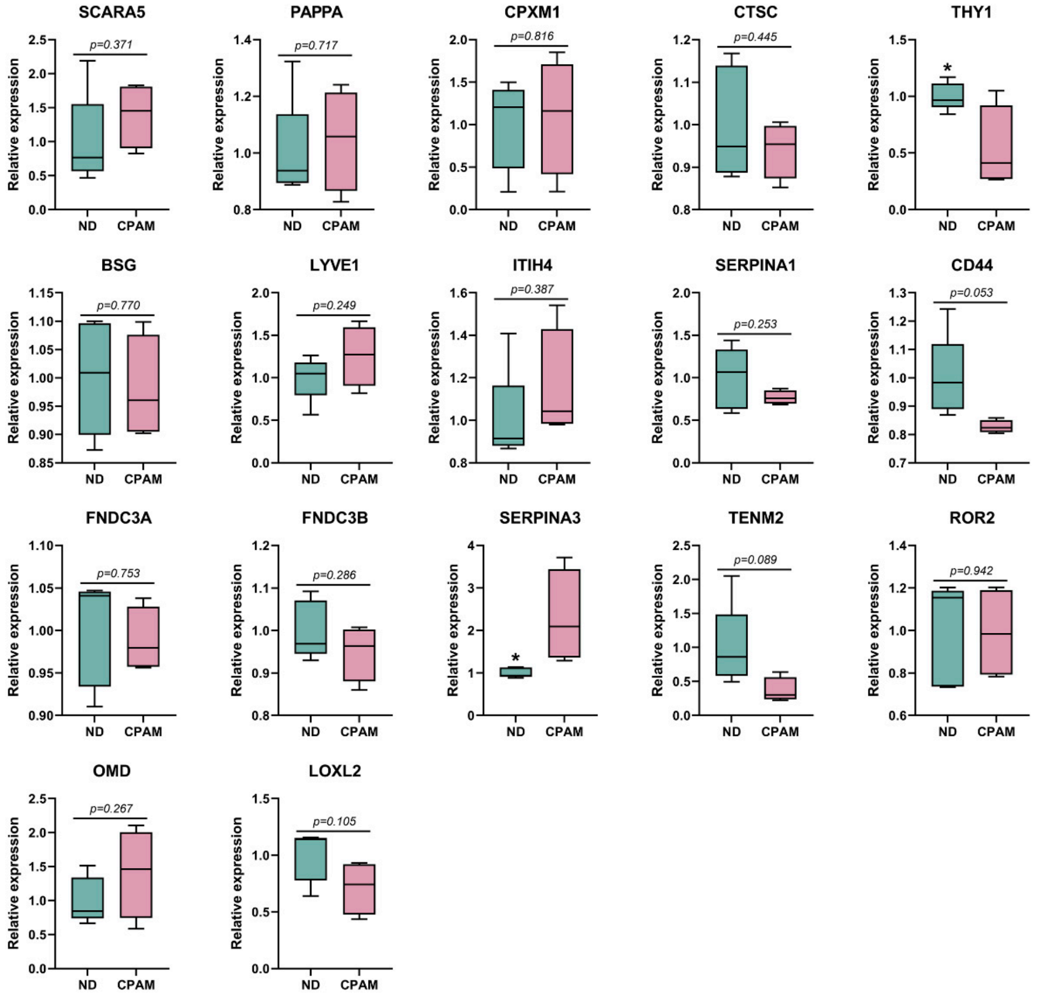


**Figure S5.** Analysis of ECM regulators from ND- and CPAM patient lungs in dataset GSE190620.

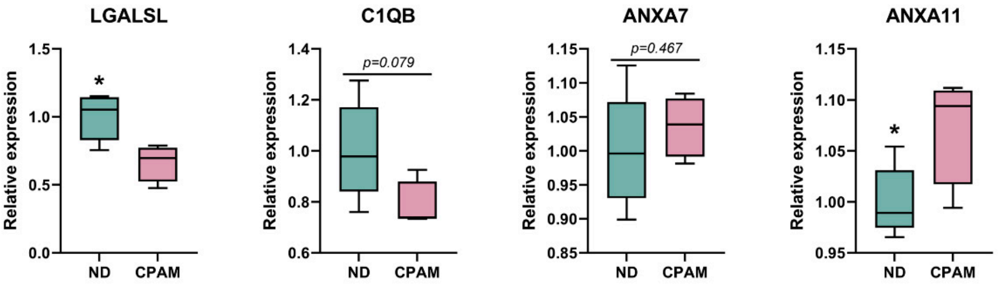


**Figure S6.** Analysis of ECM-affiliated proteins from ND- and CPAM patient lungs in dataset GSE190620.

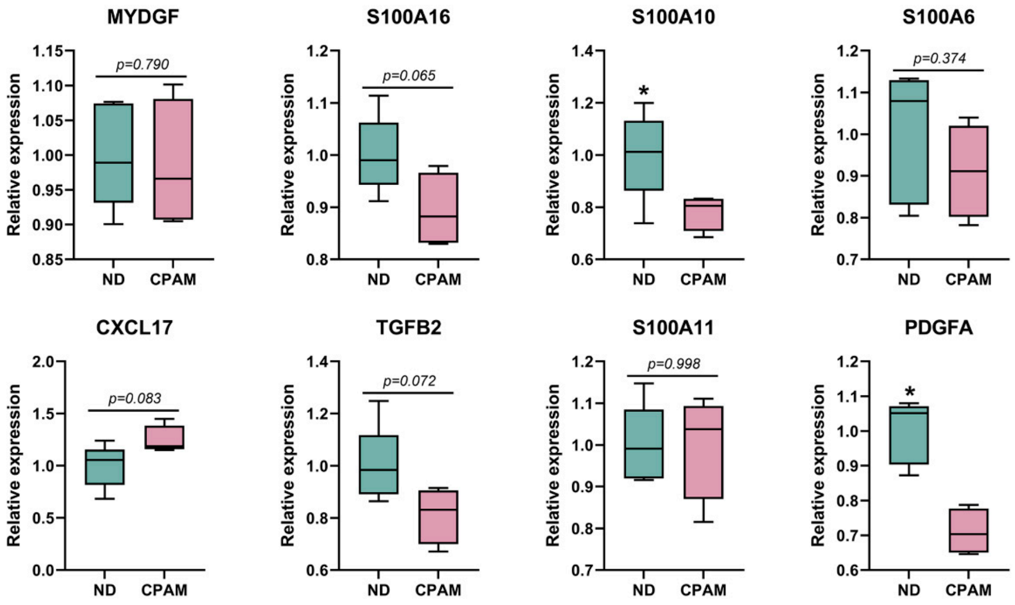


**Figure S7.** Analysis of secreted factors from ND- and CPAM patient lungs in dataset GSE190620.

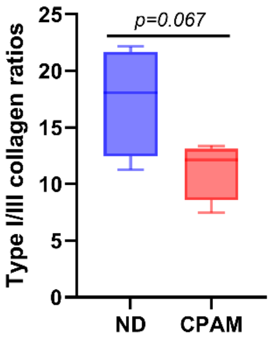


**Figure S8.** The ratios of type I/III collagen were lower in cystic areas in CPAM than in those in ND.

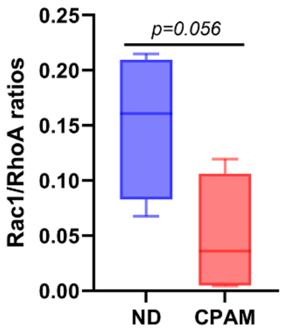


**Figure S9.** The ratios of RAC1/RHOA were lower in cystic areas in CPAM than in those in ND.
